# Fascaplysin Induces Apoptosis and Ferroptosis, and Enhances Anti-PD-1 Immunotherapy in Non-Small Cell Lung Cancer (NSCLC) by Promoting PD-L1 Expression

**DOI:** 10.3390/ijms232213774

**Published:** 2022-11-09

**Authors:** Lianxiang Luo, Guangxiang Xu

**Affiliations:** 1 The Marine Biomedical Research Institute, Guangdong Medical University, Zhanjiang 524023, China; 2 The Marine Biomedical Research Institute of Guangdong Zhanjiang, Zhanjiang 524023, China

**Keywords:** NSCLC, apoptosis, ferroptosis, ROS, PD-L1, immunotherapy

## Abstract

Fascaplysin is a natural product isolated from sponges with a wide range of anticancer activities. However, the mechanism of fascaplysin against NSCLC has not been clearly studied. In this study, fascaplysin was found to inhibit migration by regulating the wnt/β-catenin signaling pathway and reversing the epithelial–mesenchymal transition phenotype. Further research showed that the anti-NSCLC effect of fascaplysin was mainly through the induction of ferroptosis and apoptosis. Fascaplysin-induced ferroptosis in lung cancer cells, evidenced by increased levels of ROS and Fe^2+^ and downregulation of ferroptosis-associated protein and endoplasmic reticulum stress, was involved in fascaplysin-induced ferroptosis. In addition, ROS was found to mediate fascaplysin-induced apoptosis. Fascaplysin significantly upregulated the expression of PD-L1 in lung cancer cells, and enhanced anti-PD-1 antitumor efficacy in a syngeneic mouse model. Therefore, these results suggest that fascaplysin exerts anticancer effects by inducing apoptosis and ferroptosis in vitro, and improving the sensitivity of anti-PD-1 immunotherapy in vivo. Fascaplysin is a promising compound for the treatment of NSCLC.

## 1. Introduction

Lung cancer is a malignant tumor with one of the highest incidence rates in the world, of which NSCLC accounts for about 85% [1]. For a long time, the treatment methods for NSCLC were mainly surgical resection, radiotherapy, and chemotherapy, but the prognosis of patients after receiving these treatments was not good [2]. In recent years, rapid developments in immunotherapy have shown excellent efficacy in cancer treatment. Specifically, immunotherapy based on PD-1 blocking and combination therapy based on PD-1/PD-L1 blocking show potential for the treatment of NSCLC [3,4,5].

Marine-derived substances have novel structures and unique activities, and are considered a treasure trove of drugs [6]. Fascaplysin is isolated from sponges and exerts anticancer effects by inhibiting cyclin-dependent kinase 4 (CDK4), whose effects include growth inhibition, angiogenesis, metastasis, and proliferation of several cancer cells [7,8]. However, the anticancer mechanisms of fascaplysin have not been intensively studied.

Ferroptosis is an iron-dependent form of lipid peroxidative cell death [9]. Increasing evidence shows that ferroptosis is the cause or result of many pathophysiological processes in human diseases, including acute lung injury, cancer, ulcerative colitis, and so on [10]. Ferroptosis plays a key regulatory role in tumor growth and can eliminate cancer cells in an apoptosis-independent manner [11,12]. Despite the increasing research on ferroptosis, some problems remain [13]. For example, ferroptosis has certain tumor heterogeneity and gene selection characteristics, and its mechanism involves expression regulation of various genes and interacting with different signaling pathways [14]. Therefore, it is essential to study the regulatory effects and molecular mechanisms of different genes on ferroptosis in various cancer diseases, in order to find indicators that can reflect the sensitivity of cells or individuals, and target the development of novel anti-cancer drugs based on ferroptosis [15].

The spread of immune surveillance is one of the most essential features of tumorigenesis [16]. Immune checkpoint blockade (ICB) therapies that act by blocking the programmed cell death protein 1/programmed cell death ligand 1(PD-1/PD-L1) axis have shown durable antitumor activity in a variety of cancer types [2]. The clinical success of anti–PD-1/PD-L1 therapy is mainly due to the reactivation of tumor antigen-specific T cells, which are inactivated by the binding of PD-1 on T cells to its ligand PD-L1 on tumor cells [17,18]. Membrane-anchored PD-L1 has been extensively studied for its binding to PD-1 on T cells to evade antitumor immunity [19]. PD-L1 expression is tightly controlled at transcriptional and post-translational levels; however, aberrant expression of PD-L1 is observed in human cancers [16,20,21]. Gao et al proposed that high expression of PD-L1 in tumor cells would render PD-1/PD-L1 blocking inhibitors more sensitive [16,22,23]. However, the potential mechanism of how the increased expression of PD-L1 increases the sensitivity of PD-1 blockers is unclear [16]. Aguilar et al. proposed that patients with high PD-L1 expression had the greatest opportunity for clinical benefits [24]. It is suggested that patients with lung cancer with high expression of PD-L1 may benefit from the treatment of atezumab [24].

In this study, we investigated the effect of fascaplysin on the viability of lung cancer cells. We found that fascaplysin induced cell death and G1/G0 phase arrest, and inhibited the migration of lung cancer cells. Next, we demonstrated for the first time that fascaplysin induced ferroptosis in lung cancer cells; we also showed that ROS mediated the apoptosis of lung cancer cells induced by fascaplysin. In addition, we found that fascaplysin upregulated the expression of PD-L1 in vitro. Fascaplysin improved the efficacy of anti-PD-1 immunotherapy by increasing the expression of PD-L1 in vivo.

## 2. Results

### 2.1. Fascaplysin Arrested the Cell Cycle of A549 Cells by Regulating Cycle-Related Proteins

**Figure 1:**
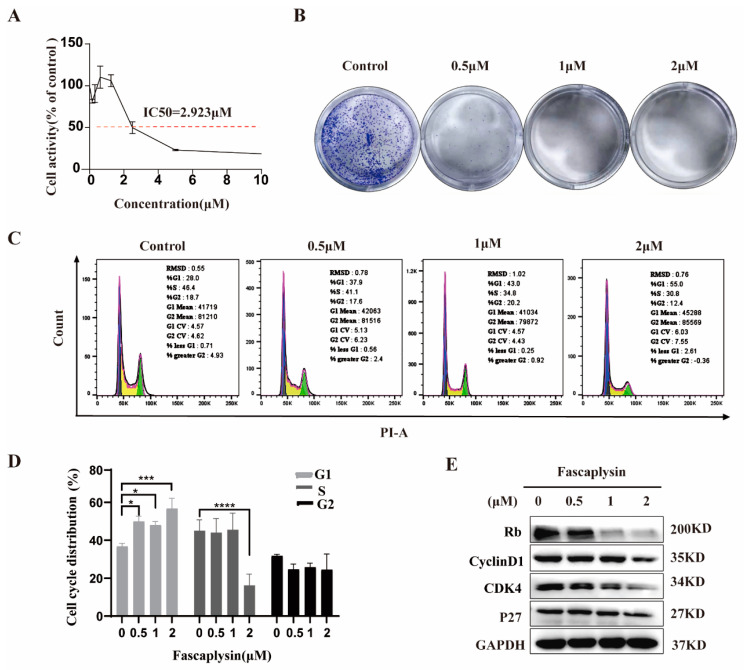
Fascaplysin arrested the cell cycle of A549 cells by regulating cycle-related proteins. (**A**) Cell viability was measured by CCK-8 assay after treatment of A549 cells with different doses of fascaplysin for 24 h. (**B**) Representative images of A549 cell colony formation after treatment with fascaplysin. (**C**,**D**) Representative results of cell cycle and quantitative analysis. Data are presented as mean ± SD of three independent experiments compared with control. * *p* < 0.05, *** *p* < 0.001, and **** *p* < 0.0001. (**E**) We examined the expression of cell cycle-related proteins by Western blotting after treating A549 cells with fascaplysin (0, 0.5, 1, and 2 μM) for 3 h.

To observe the cytotoxicity and inhibitory effect of fascaplysin on NSCLC cells, the IC50 value was 2.923 μM, as determined by CCK-8 (Figure 1A). To determine the anti-proliferative effect of fascaplysin, we performed a colony formation assay. The results showed that fascaplysin treatment significantly inhibited the colony formation efficiency of A549 cells (Figure 1B), indicating that fascaplysin inhibited the proliferation of A549 cells. To investigate whether fascaplysin reduces cell viability by altering cell cycle distribution, we performed cell cycle analysis by flow cytometry. Fascaplysin arrested the cell cycle of A549 cells in G0/G1 phase. The proportion of cells in S phase also showed a corresponding decrease (Figure 1C,D). To determine the mechanisms by which fascaplysin induces cell cycle arrest, we examined the expression of cell cycle-related proteins. Fascaplysin decreased the expression levels of cycle-related proteins Rb, cyclin D1, cyclin-dependent kinase (CDK)4, and P27 (Figure 1E). In summary, fascaplysin treatment effectively reduced the viability of A549 cells and arrested the cell cycle of A549 cells in the G0/G1 phase by regulating the expression of cell cycle-related proteins.

### 2.2. Fascaplysin Inhibited A549 Cells Migration

**Figure 2:**
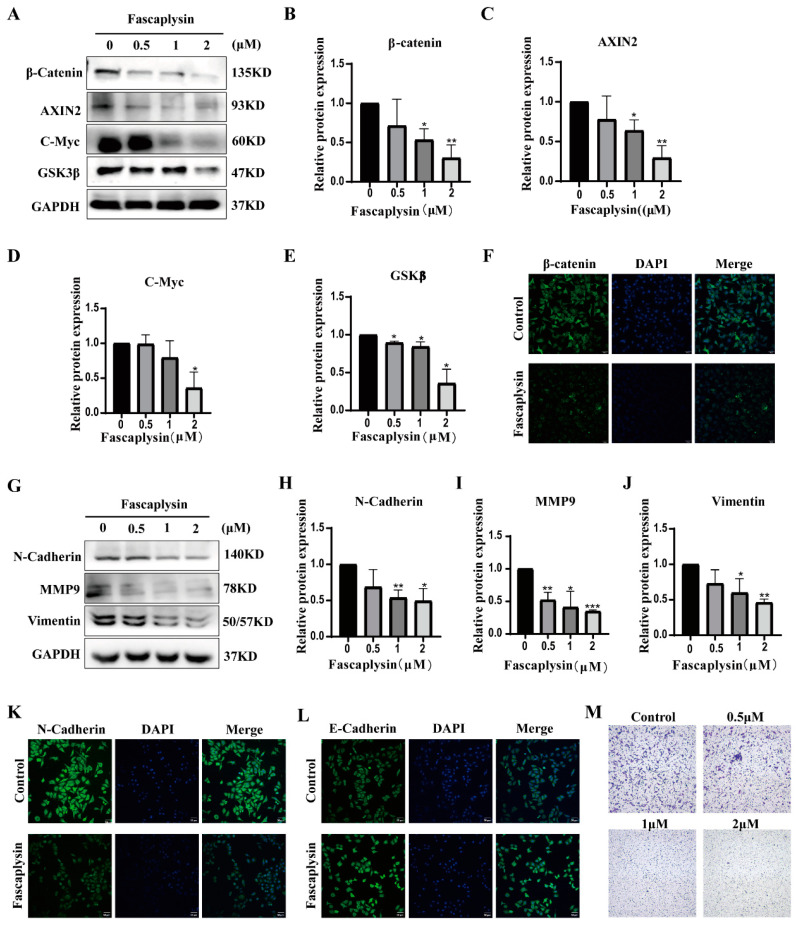
Fascaplysin inhibited A549 cells migration. (**A**) Wnt/β-Catenin signaling pathway-related proteins were detected by Western blotting. (**B**–**E**) Gray levels of Wnt/β-Catenin signaling pathway-related proteins were quantified using ImageJ and normalized to gray levels of GAPDH protein. (**F**) After A549 cells were treated with fascaplysin (2 μM) for 3 h, β-catenin protein expression levels were detected using immunofluorescence. (**G**–**J**) Detection of EMT regulatory proteins by Western blotting. (**K**,**L**) Immunofluorescence analysis of the levels of N-cadherin and E-cadherin in A549 cells. (**M**) A549 cell migration assays were performed using transwell inserts and representative images were randomly photographed. Analysis results represent mean ± SD, * *p* < 0.05, and ** *p* < 0.01, *** *p* < 0.001.

Wnt/β-catenin signaling has been found to be associated with tumor invasion and migration [25]. Wnt signaling pathway components include β-catenin, C-Myc, AXIN, and GSK3β [26]. We examined the expression of the above proteins in fascaplysin-treated A549 cells using Western blotting. The results showed that fascaplysin treatment of A549 cells decreased the expression levels of β-catenin, AXIN2, GSK3β, and C-myc proteins (Figure 2A–E). Consistent with the above results, immunofluorescence showed decreased β-catenin expression (Figure 2F).

Meanwhile, epithelial–mesenchymal transition (EMT) is considered to be closely associated with tumor progression and metastasis [27]. Therefore, we investigated whether fascaplysin inhibits cell migration by reversing the EMT phenotype. We examined the expression of EMT-related proteins in A549 cells and found that fascaplysin treatment significantly downregulated protein levels of the intercellular markers N-cadherin, vimentin, and MMP9 (Figure 2G–J). Immunofluorescence assays showed decreased N-cadherin expression and increased E-cadherin expression after fascaplysin treatment (Figure 2K,L). The effect of fascaplysin on A549 cell migration was further confirmed by a transwell assay. The results showed that fascaplysin treatment significantly inhibited A549 cell migration (Figure 2M). In summary, fascaplysin inhibited the migration of A549 cells by regulating the Wnt/β-catenin signaling pathway and reversing the EMT phenotype.

### 2.3. Fascaplysin Induced Apoptosis in A549 Cells

**Figure 3:**
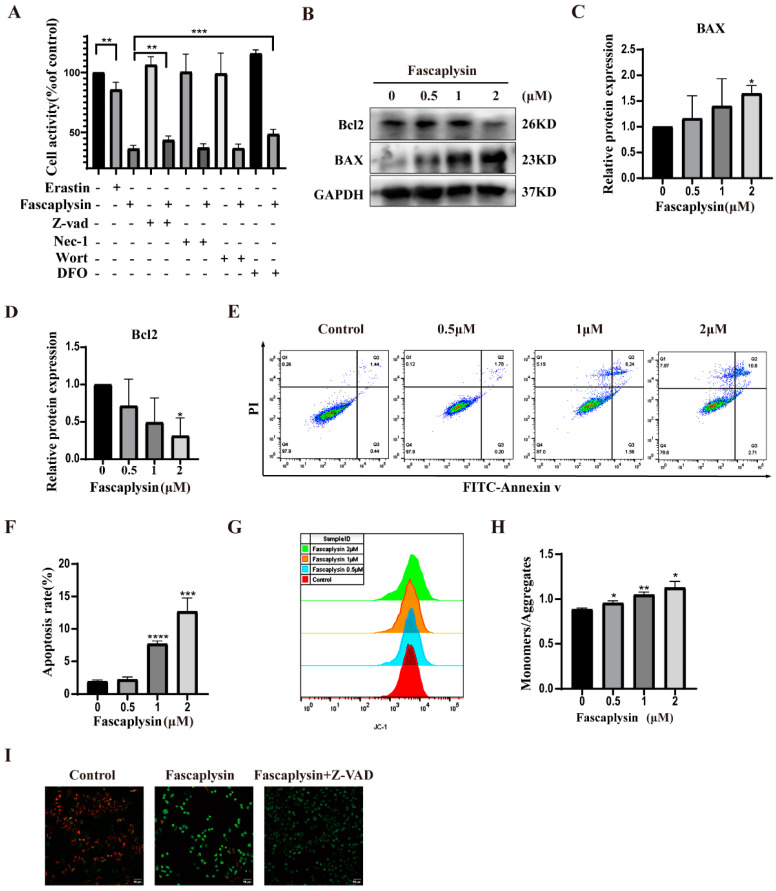
Fascaplysin induced apoptosis in A549 cells. (**A**) A549 cells were treated with erastin (10 μM), fascaplysin (2 μM), and cell death inhibitors Nec-1 (10 μM), DFO (50 μM), Z-VAD (10 μM), and Wort (5 μM), alone or in combination, for 3 h. Cell viability was measured by CCK8 assay. (**B**–**D**) BAX and Bcl2 protein expression were detected by Western blotting. (**E**,**F**) Apoptosis of fascaplysin-treated A549 cells was analyzed using flow cytometry. The histogram shows quantitative analysis of total percentage of early and late apoptotic rates. (**G**–**H**) We used JC-1 probe to examine the effect of fascaplysin on MMP in A549 cells by flow cytometry. The histogram is a statistical chart of the ratio of monomers to aggregates. (**I**) MMP was measured by confocal microscopy after treatment of A549 cells with fascaplysin (2 μM) alone or in combination with Z-VAD (10 μM). Analysis results represent mean ± SD, * *p* < 0.05, ** *p* < 0.01, *** *p* < 0.001, and **** *p* < 0.0001.

Our previous research found that fascaplysin had cytotoxic effects on A549 cells and could induce cell death. To determine the mode of cell death, we treated A549 cells with erastin, fascaplysin, an apoptosis inhibitor (Z-VAD), necrosis inhibitor (Nec-1), autophagy inhibitor (Wort), and iron ion chelator (DFO), alone or jointly, to determine the mode of fascaplysin inducing A549 cell death. The CCK8 assay showed that erastin could induce A549 cell death, and the apoptosis inhibitor (Z-VAD) and iron ion chelator (DFO) could rescue fascaplysin-induced cell death (Figure 3A). It was preliminarily determined that fascaplysin could induce apoptosis of A549 cells. The iron chelating agent DFO protected against the cytotoxicity of fascaplysin, indicating that fascaplysin-induced cell death is iron-dependent.

To determine whether fascaplysin could induce apoptosis in A549 cells, we used flow cytometry after double staining with Annexin V-FITC and PI. The results showed that the apoptosis rate of A549 cells increased with an increase in fascaplysin concentrations (Figure 3E,F). Studies have reported that Bcl2 family members play an important role in apoptosis by regulating mitochondrial membrane permeability [28]. Bcl2 is an inhibitor of apoptosis proteins and BAX is an apoptosis-promoting protein [29]. We examined the expression of Bcl2 and BAX proteins by Western blotting, and the results showed that the expression of Bcl2 tended to decrease, and BAX tended to be significantly upregulated (Figure 3B–D). Apoptosis is a process of programmed cell death, and mitochondrial apoptosis-inducing channels are early markers of apoptosis development [30]. There is a difference in the voltage across the mitochondrial membrane, called the mitochondrial membrane potential (MMP or Δψ) [30]. Significant loss of ΔΨm causes energy depletion and cell death [31]. We examined the effect of fascaplysin on the mitochondrial membrane potential in A549 cells using a JC-1 probe. The results showed that the mitochondrial membrane potential of A549 cells decreased significantly after fascaplysin treatment, and this decrease in mitochondrial membrane potential was attenuated after fascaplysin co-treatment with Z-VAD (Figure 3G–I). In summary, fascaplysin induced apoptosis and caused a decrease in the mitochondrial membrane potential of A549 cells.

### 2.4. Fascaplysin Induced Ferroptosis and Endoplasmic Reticulum Stress (ER Stress) in A549 Cells

**Figure 4:**
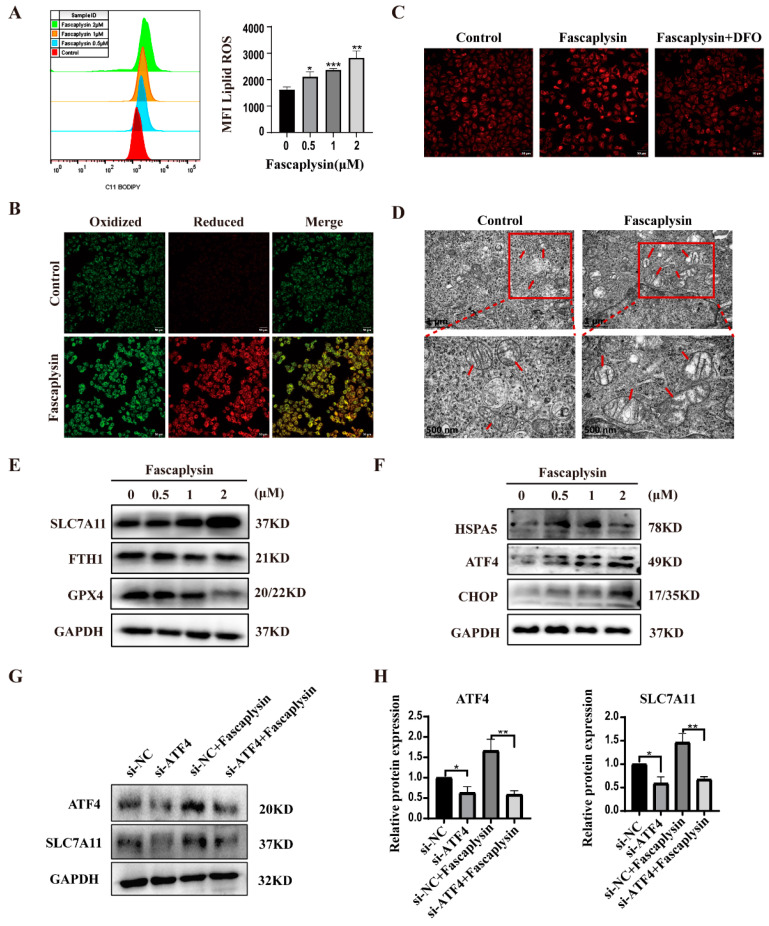
Fascaplysin induced ferroptosis and ER stress in A549 cells. (**A**) Representative images and statistical results of lipid ROS in A549 cells. (**B**) Fluorescence images of lipid peroxidation in A549 cells were observed using a C11-BODIPY-581/591 probe. (**C**) A549 cells were treated with fascaplysin (2 μM) and DFO (50 μM) for 3 h, and fluorescence images of cellular Fe^2+^ were detected using a Ferrorange probe. (**D**) Intracellular mitochondrial morphological changes in A549 cells were observed by transmission electron microscopy (TEM) (original magnification: 6000×, 80,000×). Red arrows indicate mitochondria (**E**) Ferroptosis-related proteins were detected by Western blotting. (**F**) ER stress-related proteins were detected by Western blotting. (**G**,**H**) A549 cells were transfected with si-ATF4 (20 nM) for 60 h and treated with fascaplysin for 3 h. ATF4 and SLC7A11 protein expression was then detected by Western blotting. Analysis results represent mean ± SD, * *p* < 0.05, ** *p* < 0.01, and *** *p* < 0.001.

Ferroptosis is a novel oxidatively regulated mode of cell death (RCD) driven by iron-dependent lipid peroxidation [32]. Our previous study found that fascaplysin and the iron ion chelator DFO could save A549 from cell death after co-treatment, and the classical ferroptosis inducer erastin could induce A549 cell death (Figure 3A). Thus, we expected fascaplysin to induce A549 cell ferroptosis. Next, we examined intracellular lipid ROS, Fe^2+^ levels, and changes in mitochondrial morphology and other ferroptosis-related parameters. As we expected, intracellular lipid ROS significantly rose in A549 cells treated with fascaplysin (Figure 4A,B). Iron is an essential active element for a variety of biological processes, including ferroptosis [33]. We used Ferrorange (a specific probe for Fe^2+^) to determine intracellular Fe^2+^ levels. The results showed that the intracellular red fluorescence signal was significantly enhanced after fascaplysin treatment, and significantly decreased after co-treatment with DFO (Figure 4C). Observation of mitochondrial morphology with transmission electron microscopy revealed increased mitochondrial membrane density and atrophy or disappearance of cristae (Figure 4D).

It is well-known that GPX4 is a classical pathway for ferroptosis [34]. GPX4 is a selenocysteine-containing enzyme that breaks down lipid peroxides, preventing iron-mediated peroxide reactions, leading to ferroptosis [35]. Glutathione mediates the recycling process of GPX4. Glutathione in cells is synthesized from cysteine and requires the Xc^-^ system (XcT) to transport cystine into cells; SLC7A11 is a multichannel transmembrane protein on the XcT [36]. It has been shown that ferritin is also a key ferroptosis regulator [37]. We examined GPX4, FTH1, and SLC7A11 protein expression by Western blotting, and the results showed that GPX4 and FTH1 protein expression was downregulated and SLC7A11 protein expression was upregulated (Figure 4E). Studies have shown that ER stress response is synchronously activated with the ferroptosis induced by ferroptosis inducers in cancer cells [38]. We speculated that abnormal SLC7A11 protein expression may be associated with ER stress. ER stress is a biological state characterized by the expansion or misfolding of proteins [39]. Elevated expression levels of activated transcription factors (ATFs), such as ATF4, form a feedback loop that ultimately regulates ferroptosis through transcriptional activation of several downstream genes [40]. It has been shown that transcriptional activator 4 (ATF4) is a key mediator of metabolic and oxidative homeostasis and cell survival and that ATF4 expression is elevated by various microenvironmental stresses, including starvation, ER stress, and exposure to toxic factors [41]. ATF4-dependent tumor promotion is mediated by transcriptional targeting of the glutamate reverse transporter Xc/SLC7A11 [41]. Thus, XcT was elevated due to ATF4 activation [41]. It has also been shown that ATF4 can play a dual role in inhibiting or promoting ferroptosis by upregulating SLC7A11, HSPA5, and CHOP [40]. We examined the associated proteins by Western blotting, and the results showed that ATF4, HSPA5, and CHOP expressions were significantly upregulated in fascaplysin-treated cells (Figure 4F). To determine whether upregulation of SLC7A11 was caused by ER stress, we knocked down ATF4 in A549 cells using a si-ATF4 transfection reagent and then examined the effect on SLC7A11 protein expression. The results showed that the expression of SLC7A11 protein was downregulated after knocking down ATF4 (Figure 4G–H).

The above results showed that fascaplysin could induce ferroptosis and cause ER stress in A549 cells, and that ER stress mediates ferroptosis.

### 2.5. ROS Played an Important Role in Fascaplysin-Induced Apoptosis and Ferroptosis in A549 Cells

**Figure 5:**
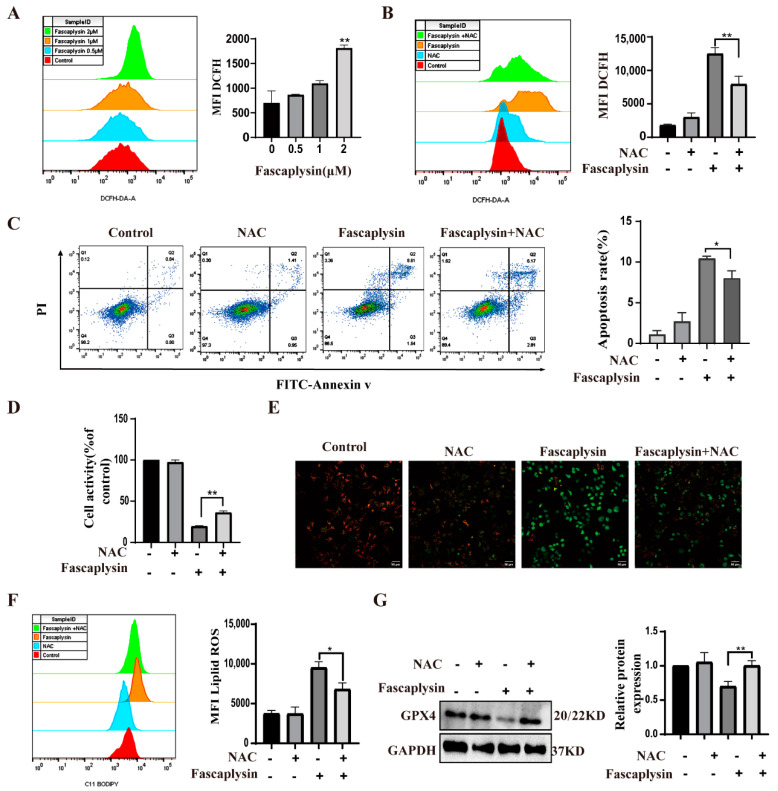
ROS played an important role in fascaplysin-induced apoptosis in A549 cells. (**A**) ROS in A549 cells was detected by flow cytometry with DCFH−DA probe. (**B**) ROS was detected by flow cytometry after treatment of A549 cells with the NAC (1 mM) and fascaplysin (2 μM), alone or in combination, for 3 h. (**C**) A549 cells were treated with NAC and fascaplysin, alone or in combination, and then apoptosis was detected by flow cytometry after double staining with annexin V-FITC/PI. (**D**) Cell viability was measured by CCK-8. (**E**) MMP changes were analyzed with a JC-1 probe. (**F**) A549 cells were treated with fascaplysin and NAC, alone or in combination, and lipid ROS levels were measured with a C11 BODIPY probe. (**G**) A549 cells were treated with fascaplysin and NAC, alone or in combination, and GPX4 protein expression levels were detected by Western blotting. Analysis results represent mean ± SD, * *p* < 0.05, ** *p* < 0.01.

Many studies have shown that oxidative stress (ROS) can lead to apoptosis [42]. ROS are reactive oxygen species that can activate mitochondria-dependent apoptosis [30]. We investigated whether fascaplysin-induced apoptosis in A549 cells resulted from elevated ROS. We detected ROS by flow cytometry. The results showed that fascaplysin dose-dependently increased intracellular ROS in A549 cells (Figure 5A). N-acetyl-l-cysteine (NAC), as a conventional antioxidant, scavenges intracellular ROS [43]. We examined ROS after treating A549 cells with a combination of f and NAC and found that NAC significantly decreased ROS levels and apoptotic rates, whereas NAC itself had no significant effect on intracellular ROS and apoptotic rates in A549 cells (Figure 5B,C). Next, cell viability was measured by CCK8 assay, and the results were consistent with the above (Figure 5D). The above results suggest that the accumulation of intracellular ROS has an important effect on fascaplysin-induced apoptosis in A549 cells. To further understand the mechanism of ROS in fascaplysin-induced apoptosis in A549 cells, we investigated the effect of ROS on mitochondrial membrane potential in A549 cells. After treatment with NAC, ROS levels decreased and MMP loss was significantly alleviated (Figure 5E). These findings suggested that ROS mediated fascaplysin-induced apoptosis. We also tested the effect of ROS on ferroptosis. Compared with A549 cells treated with fascaplysin alone, the level of lipid reactive oxygen species (ROS) decreased and expression of GPX4 protein increased when fascaplysin and NAC were treated together. These results indicated that ROS also played an important role in fascaplysin-induced ferroptosis (Figure 5F,G).

### 2.6. Fascaplysin Promoted PD-L1 Expression in A549 Cells

**Figure 6:**
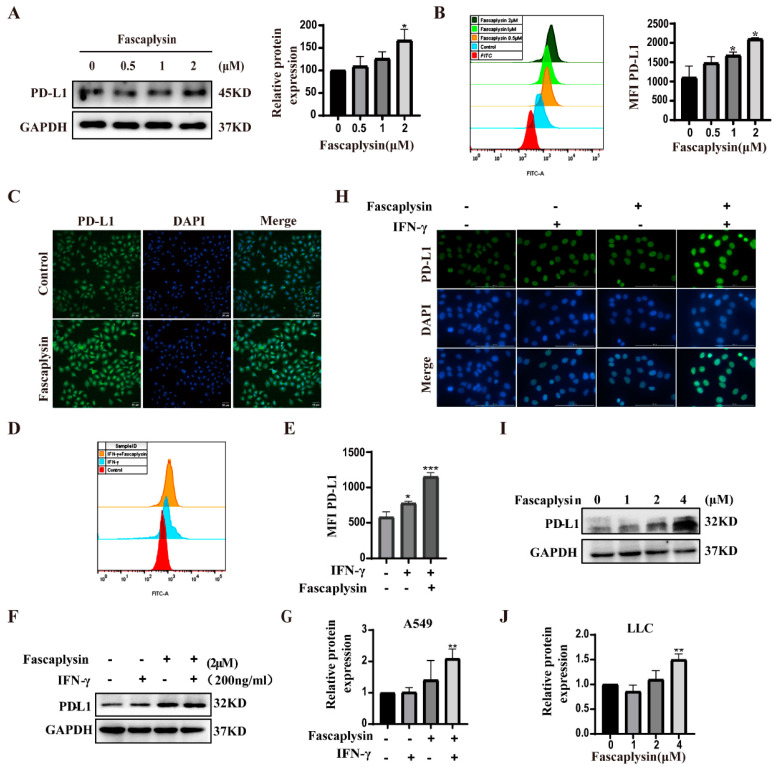
Fascaplysin promoted PD-L1 expression in NSCLC cells. (**A**) PD-L1 protein expression was detected by Western blotting. (**B**) After treatment of A549 cells with the PD-L1 kit, the fluorescence intensity of PD-L1 was analyzed by flow cytometry. The histogram is a quantification of the fluorescence intensity of PD-L1. (**C**) Immunofluorescence showing the level of PD-L1 protein in A549 cells. (**D**,**E**) A549 cells were treated with IFN-γ (200 ng/mL) and fascaplysin (2 μM) for 3 h and PD-L1 fluorescence intensity was detected by flow cytometry using a PD-L1 kit. Histograms show quantitation of PD-L1 fluorescence intensity. (**F**,**G**) A549 cells were treated with fascaplysin and IFN-γ, alone or in combination, and PD-L1 expression was examined by Western blotting. (**H**) LLC cells treated with fascaplysin (4 μM) and IFN-γ (200 ng/mL), alone or in combination, were examined for PD-L1 expression by immunofluorescence. (**I**,**J**) LLC cells were treated with fascaplysin (0, 1, 2, 4 μM) at various concentrations for 3 h to detect PD-L1 expression by Western blotting. Analysis results represent mean ± SD, * *p* < 0.05, ** *p* < 0.01, *** *p* < 0.001.

Programmed death ligand 1 (PD-L1) is a cell surface protein that inhibits the killing function of T cells by interacting with PD-1 receptors on the surface of activated T and B cells and negatively regulates the human immune response, no longer attacking tumor cells [44]. It has been shown that, in some solid tumors, the response rate against PD-L1 is limited [45]. Therefore, further understanding of the regulatory mechanisms of PD-L1 can bring substantial benefits to cancer patients by improving the efficacy of current PD-L1/PD-1 blockers or other ICBs [46]. We investigated the effect of fascaplysin on PD-L1 expression in A549 cells, and Western blot results showed that fascaplysin upregulated PD-L1 protein expression (Figure 6A). Immunofluorescence analysis and flow cytometry analysis showed that fascaplysin could enhance the fluorescence intensity of PD-L1 (Figure 6B,C). Studies have shown that IFN-γ is mainly produced by activated T cells, and IFN-γ can promote the expression of PD-L1. We examined the expression of PD-L1 by flow cytometry after combined treatment with IFN-γ and fascaplysin, and the results suggested that the expression of PD-L1 was significantly increased in the combined treatment group (Figure 6D–G). We also examined PD-L1 expression levels in LLC cells. The expression level of PD-L1 was significantly increased after treatment with the combination of fascaplysin and IFN-γ (Figure 6H). The expression of PD-L1 protein in LLC cells was upregulated by treatment with fascaplysin at different concentration gradients (Figure 6I,J). The above results indicated that fascaplysin could promote PD-L1 expression in A549 and LLC cells.

### 2.7. Protein Ligand Interaction Energy Analysis

**Figure 7:**
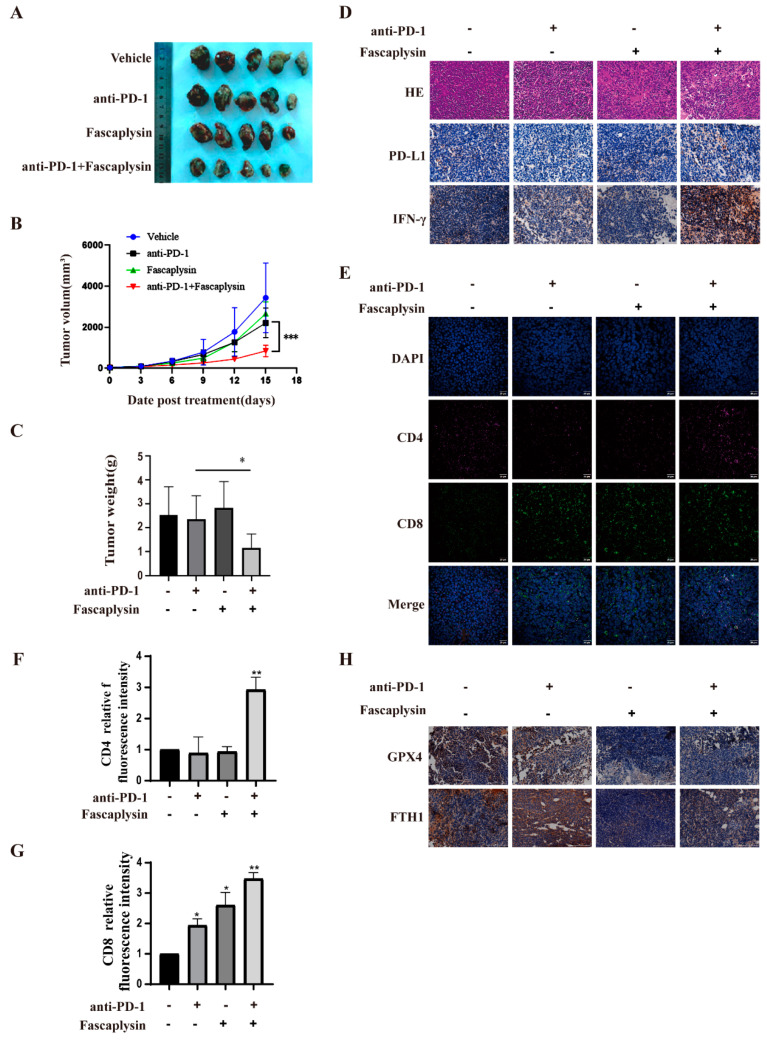
Fascaplysin enhanced the efficacy of PD-1 checkpoint blockade. (**A**) Images of tumor tissue excised from LLC tumor-bearing mice after treatment with fascaplysin and anti-PD-1. (**B**) Growth curves of tumor volumes in each group. (**C**) The tumor weight of each group was counted. (**D**) Paraffin sections of tumor tissues were analyzed by HE staining; the expression of PD-L1 and IFN-γ in tumor tissues was detected by IHC. (Scale bar, 100 μm). (**E**–**G**) Representative images of CD4^+^ (red) or CD8^+^ (green) and DAPI nuclear staining (blue) in tumor tissues detected by multiplex immunofluorescence staining technique (Magnification: oil immersion lens 60×); quantitative analysis was carried out using Image J software. (**H**) IHC was used to detect the expression of GPX4 and FTH1 in tumor tissues (Scale bar, 100 μm.). Analysis results represent mean ± SD, * *p* < 0.05, ** *p* < 0.01,*** *p* < 0.001.

At present, numerous studies have shown that blocking the PD-L1/PD-1 pathway shows significant anti-tumor effects in patients with advanced cancer, and is considered to be the standard for the development of the new immune checkpoint blockade (ICB) and combination therapy [46]. Based on the cell experiments described above, we investigated the antitumor effect of fascaplysin combined with anti-PD-1 in vivo. We treated LLC tumor-bearing mice with vehicle, fascaplysin, anti-PD-1, or fascaplysin in combination with anti-PD-1 for 15 days. The tumor size gradually increased in the control group, fascaplysin treatment group, and anti-PD-1 treatment group. However, tumor size was reduced in the group treated with the combination of fascaplysin and anti-PD-1 (Figure 7A). Next, we counted the volume and weight of tumor tissue and found that the combination of fascaplysin and anti-PD-1 had statistically significant reductions compared with the control group (Figure 7B,C). In addition, HampE staining of tumor tissue sections also showed that fascaplysin combined with anti-PD-1 significantly increased the tumor necrosis area compared with the other treatment groups (Figure 7D). The above results suggested that the combination treatment of fascaplysin with anti-PD-1 significantly inhibited the growth of transplanted tumors in mice.

We investigated the infiltration of CD4^+^ and CD8^+^ T cells in tumor tissues to characterize the effect of the combination of fascaplysin and anti−PD−1 on the immune response against tumors in vivo. Multiplex immunofluorescence staining showed that CD4^+^ and CD8^+^ T cell infiltration was significantly increased in the combination group compared with the monotherapy group (Figure 7E–G). In addition, immunohistochemical staining showed that the expression of PD-L1 and IFN-γ in the combined treatment group was significantly higher than in the monotherapy group (Figure 7D). In addition, immunohistochemical staining showed that the expression of GPX4 and FTH1 in the fascaplysin alone and combined treatment groups was lower than that in the control and anti-PD-1 treatment groups. The above results indicated that fascaplysin could induce ferroptosis in vivo (Figure 7H). These results suggest that the combination of fascaplysin and anti-PD-1 can promote an anti-tumor immune response and inhibit the growth of NSCLC by promoting the expression of PD-L1. Moreover, our results also showed that fascaplysin could induce ferroptosis in NSCLC.

## 3. Discussion

**Figure 8:**
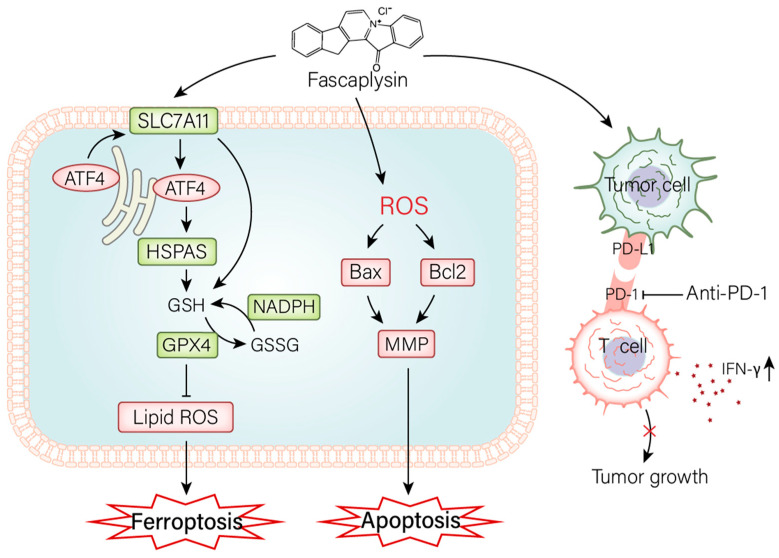
The mechanism of fascaplysin inhibiting the growth of NSCLC in vitro and in vivo. In vitro, fascaplysin induced apoptosis by promoting the elevation of ROS. It induced ferroptosis by modulating the GPX4 signaling pathway and provoked ER stress. In vivo, fascaplysin improved the sensitivity of anti-PD-1 immunotherapy, which in turn inhibited tumor growth.

Previous studies have shown that marine-derived compound fascaplysin has biological activity against a variety of cancer cells. However, there are not many studies on the specific mechanism of action of fascaplysin against cancer. This study investigated the effect of fascaplysin on NSCLC in vivo and in vitro (Figure 8). The results showed that fascaplysin promoted cell cycle G0/G1 arrest, inhibited cell migration, induced apoptosis and ferroptosis, and promoted PD-L1 expression in vitro. Fascaplysin improved the sensitivity of anti-PD-1 immunotherapy by promoting PD-L1 expression in vivo.

Programmed cell death is an active, orderly, and genetically controlled death process, including apoptosis, necrosis, pyrosis, autophagy, and ferroptosis [47]. It plays an important role in the occurrence, development, and treatment of tumors [47]. In this study, we found that fascaplysin induced NSCLC cell death by apoptosis and ferroptosis, and explored the mechanisms involved. First, we found that fascaplysin downregulated the expression of Bcl2 and upregulated the expression of BAX, and that the apoptosis inhibitor Z-VAD effectively attenuated the decrease in mitochondrial membrane potential. These results confirmed that fascaplysin-induced apoptosis is a mitochondrial apoptotic pathway in A549 cells. We also found that NAC could inhibit fascaplysin-induced elevation of ROS, increase apoptosis rate, and decrease MMP. These results confirmed that ROS plays an important role in the mitochondrial apoptosis pathway in A549 cells, and indicated that ROS accumulation plays an upstream role in fascaplysin-induced apoptosis. However, the mitochondrial pathway was not completely blocked by the antioxidant NAC, so we suspect that this pathway is also affected by other factors that are not affected by ROS [48]. The mechanism by which fascaplysin induces ROS production is unknown. The sources of intracellular ROS include peroxisomes, NADPH oxidase, and the mitochondrial electron transport chain [49].

The aim of traditional cancer therapy is to induce apoptosis, but many cancer cells are resistant to chemotherapy or defective in inducing apoptosis [33]. Therefore, the development of new drugs that enhance different forms of non-apoptotic cell death is expected to provide a promising therapeutic strategy for cancer patients [33]. In recent years, great progress has been made in the study of novel anticancer drugs induced by ferroptosis [10]. In this study, we found for the first time that fascaplysin was able to induce ferroptosis in A549 cells by increasing intracellular lipid ROS, increasing Fe^2+^ levels, regulating proteins such as GPX4, FTH1, and SLC7A11, and activating the ER stress response. Our study results suggest that ER stress is involved in ferroptosis. Recently, an increasing number of studies have demonstrated that ferroptosis is a promising therapeutic target [50]. Therefore, further understanding the relationship between fascaplysin-induced ferroptosis and ER response is important for the development of fascaplysin as a clinically efficient drug against NSCLC [51].

PD-L1 expression is the most useful predictive biomarker for the efficacy of immunotherapy in NSCLC, and CD8^+^ T cells play a crucial role in the clinical activity of immunotherapy [52]. Immune checkpoint inhibitors against PD-1 or PD-L1 have significantly improved the prognosis of patients with a variety of cancers, although only 20–40% of patients benefit from these new therapies [53]. PD-L1, quantified by immunohistochemical analysis, is currently the most widely validated, used, and accepted biomarker to guide patient selection for anti-PD-1 or anti-PD-L1 antibodies [54]. Immunotherapy based on PD-1 blockade has revolutionized the prognosis of patients with non-small cell lung cancer [55]. In our study, in vitro studies showed that fascaplysin promoted the expression of PD-L1, and in vivo combination therapy of fascaplysin and anti-PD-1 showed strong anticancer effects, whereas treatment alone had no significant anticancer effects. The detection of relevant parameters in tumor tissues revealed increased PD-L1 and IFN-γ expression and enhanced tumor-infiltrating CD8^+^ and CD4^+^ T in the fascaplysin and anti-PD-1 combination therapy groups. Our study finally demonstrated that fascaplysin enhanced the sensitivity of anti-PD-1 immunotherapy by promoting PD-L1 expression. It is well-known that T cells can kill tumors and release IFN-γ. Studies have reported that IFN-γ has an inhibitory effect on established tumors in anti-tumor immune responses [56]. Moreover, our study also showed that the combination of fascaplysin and IFN-γ could promote the expression of PD-L1; thus, we speculate that this interaction plays an important role in the strong anticancer effect of combination therapy of fascaplysin and anti-PD-1.

## 4. Materials and Methods

### 4.1. Cell Culture

A549 and LLC cell lines were purchased from the American Type Culture Collection (ATCC). A549 cells were cultured in a RPMI-1640 medium containing 10% fetal bovine serum (Gibco, Grand Island, NE, USA) and 1% penicillin-streptomycin (Gibco, Grand Island, NE, USA). LLC cells were cultured in a DMEM medium containing 10% fetal bovine serum (Gibco, Grand Island, NE, USA) and 1% penicillin-streptomycin. They were incubated at 37 °C and 5% CO_2_.

### 4.2. Antibodies and Reagents

Cell lysates were prepared by adding PMSF with RIPA lysate (Solarbio, Beijing, China), followed by protein quantification using a BCA protein assay kit (Sangon Biotech, Shanghai, China). Proteins were subsequently separated with 12% SDS-PAGE and transferred to nitrocellulose membranes. The membrane was blocked with 5% bovine serum albumin (BSA), and then the membrane was incubated with anti-GPX4 (CST, Shanghai, China), FTH1 (CST, Shanghai, China), SLC7A11 (ABclonal, Wuhan, China), GAPDH (Sangon Biotech, Shanghai, China), PD-L1 (CST, Shanghai, China), Vimentin (ABclonal, Wuhan, China), C-Myc (Wanleibio, Wuhan, China), AXIN2 (ABclonal, Wuhan, China), GSK3β (Wanleibio, Wuhan, China), β-Catenin (CST, Shanghai, China), MMP9 (ABclonal, Wuhan, China), ATF4 (ABclonal, Wuhan, China), HSPA5 (CST, Shanghai, China), CHOP (Wanleibio, Wuhan, China), BCL2 (Wanleibio, Wuhan, China), BAX (Wanleibio, Wuhan, China), Cyclin D (CST, China), CDK4 (Wuhan, China), and P27 (Wanleibio, Wuhan, China). The next day, the membranes were incubated with HRP-linked secondary antibodies (1:4000; CST, Shanghai, China) for 1 h at room temperature. Finally, color development was performed using BeyoECL Moon (Beyotime Biotechnology, Shanghai, China). The antibodies used in the immunofluorescence assay were E-cadherin (Wanleibio, Wuhan, China), N-cadherin (Wanleibio, Wuhan, China), and IFN-γ (Wanleibio, Wuhan, China). The inhibitors mentioned in the experiments were deferoxamine (Cat: S5742) (Selleck, Houston, United States), Fer-1 (Cat: 57243) (Sigma-Aldrich, St. Louis, United States), Z-VAD-FMK (Cat: HY-16658B), n-acetylcysteine (Cat: HY-B0215), necrostatabsin-1 (Cat: HY-15760), and Wort (Cat 817875).

### 4.3. Cytotoxicity Assays and Clone Formation Assays

Cell counting kit-8 (CCK8) (Beyotime Biotechnology, Shanghai, China) was used for cytotoxicity analysis. Cells (5 × 10^3^/well) were plated in 96-well plates and stimulated with drugs at different concentration gradients for 24 h. CCK-8 solution was added and incubated in an incubator for 3 h. The absorbance value was measured at 450 nm, and Cancer Chemopreventative Properties (IC50) as calculated via GraphPad Pro Prism8.0 (GraphPad, San Diego, CA, USA). As for colony formation assay, cells (1 × 10^3^/well) were plated in 12-well plates and cultured for 24 h. Stimulation with different drug concentrations was added and cultured for 7 days. Cloned cell colonies were fixed, stained, and imaged.

### 4.4. Apoptosis Assays

Cells (1 × 10^5^ cells/well) were plated in 12-well plates for 24 h. Cells were collected after drug stimulation. They were stained for 15 min using the Annexin V/FITC Apoptosis Detection Kit (Cat: 556547) (BD, Pharmingen, San Diego, CA, USA) in the dark. The specific treatment process was performed according to the manufacturer’s instructions. Analysis was performed on a flow cytometer (BD FACSCelesta, East Rutherford, NJ, USA) within 1 h.

### 4.5. Mitochondrial Membrane Potential Assay

After the cells were plated and treated with drug stimulation, the mitochondrial membrane potential was measured using the mitochondrial membrane potential assay JC-1 dose box (Beyotime Institute, Jiangsu, Nanjing, China). The specific treatment process was performed according to the manufacturer’s instructions. The processed samples were assayed for mitochondrial membrane potential using confocal microscopy (FV300, OLYMPUS, JapanOLYMPUS, 1xplore SpinsR).

### 4.6. Cell Migration Assays

The migration ability of A549 cells was assessed using a transwell cell culture chamber, and 200 μL of A549 cells (5 × 10^4^) were seeded into the upper chamber for 24 h. The upper chamber was replaced with a serum-free medium containing different concentrations of fascaplysin, and the lower chamber was supplemented with 700 μL of medium with 10% fetal bovine serum. After 16 h of incubation in the incubator, they were fixed in 4% paraformaldehyde and stained with 0.5% crystal violet for 15 min. After washing five times with PBS, other cells on the upper surface were carefully wiped. Then, imaging was performed with a microscope (×4 magnification).

### 4.7. Measurement of Ferrous Ion

Cells were plated and treated with drug stimulation, and the medium containing the drug in the well was discarded and replaced with a serum-free medium containing 1 µM Ferro Orange (Dojindo, Japan), and incubation was continued for 30 min at 37 °C and 5% CO_2_ in the dark. Then, photographs were observed under a confocal microscope.

### 4.8. Determination of ROS and Lipid ROS

For ROS analysis, cells collected by trypsin digestion were incubated with 2 μM 2′,7′-dichlorofluorescein diacetate (DCFH-DA) (ABclonal, Wuhan, China) for 20 min at 37 °C in the dark, and then the cells were washed twice with a serum-free medium and analyzed on a flow cytometer. For analysis of lipid ROS, C11-BODIPY 10 µM (ABclonal, Wuhan, China) was added to A549 cells for 1 h at 37 °C, 5% CO_2_, and the cells were washed twice with PBS. Then, cells were digested with trypsin, suspended in PBS containing 5% FBS, and analyzed on a flow cytometer. Sample processing was consistent with the flow cytometry pre-processing step, using confocal microscopy to measure the fluorescence intensity of C11 BODIPY displaying ROS in cells. The fluorescence intensity was photographed and observed at excitation wavelengths of 488 and 561 nm.

### 4.9. Cell Cycle Staining Assay

For cell cycle analysis, drug-treated cells were collected, centrifuged, and washed twice. Then, cells were fixed with 75% cold ethanol overnight. After washing twice with PBS to remove residual ethanol, cells were then left in the dark for 15 min in a complex with RNase A (100 μg/mL), propidium iodide (PI; 50 μg/mL), and 0.2% Triton X-100. Finally, the distribution of cells in different cell cycles was examined using flow cytometry.

### 4.10. Immunofluorescence Assay

A549 cells were seeded in 6-well plates with slides. After exposure to the drug for 3 h, the cells were fixed for 20 min, followed by disruption of the cell membrane using 0.5% Triton X-100 for 30 min. Subsequently, cells were blocked using 5% goat serum before incubation with primary antibodies to the relevant proteins overnight at 4 °C. Cells were washed three times the next day and then cultured for 1 h at room temperature using Alexa Fluor 488 secondary antibodies. Finally, nuclei were stained with DAPI buffer, and then cells were photographed with confocal microscopy.

### 4.11. Western Blotting Analysis

Cells were seeded in 12-well plates, collected after 3 h of stimulation with fascaplysin, and then total protein was extracted using radioimmunoprecipitation assay (RIPA) lysis buffer containing protease and phosphatase inhibitors (Sigma-Aldrich). Total proteins were separated using SDS-PAGE (8–12%) and transferred to NC membranes. Membranes were then blocked using 5% BSA for 1 h at room temperature and incubated overnight at 4 °C using specific primary antibodies. After these membranes were washed three times with TBST buffer, they were incubated for 1 h at room temperature using the corresponding secondary antibodies. Then, protein bands were detected using a chemiluminescence kit (Millipore). GAPDH was used as an internal reference.

### 4.12. Transfection

Si-ATF4 and si-NC (Sangon Biotech, Shanghai, China) were each dissolved and prepared according to the instructions. After equilibration for 12 min at room temperature, the siRNA liposomes were gently mixed and allowed to form. A549 cells were transfected with transfection mix in an antibiotic-free cell culture medium.

### 4.13. Mouse Models

The experiment was approved by the Animal Center Care and Use Committee of Guangdong Medical University. LLC cells (5 × 10^6^ cells/100 μL) were subcutaneously injected into the right dorsal side of 6-week-old male C57BL/6 mice (Yison BIO Shanghai, China). When the average tumor volume reached 70–100 mm^3^, LLC tumor-bearing mice were randomly divided into 4 groups (5 mice/group): (1) control group, (2) fascaplysin group, (3) anti-PD-1 group, and (4) fascaplysin combined with anti-PD-1 treatment group. LLC tumor-bearing mice were treated with fascaplysin in 3% dimethyl sulfoxide phosphate-buffered saline, and the control group received the same amount of the corresponding diluent. LLC tumor-bearing mice received intraperitoneal injections of fascaplysin (10 mg/kg/day) and anti-PD-1 (10 mg/kg/3 days). After 15 days of treatment, mice were sacrificed by cervical dislocation and tumor tissue was removed. Tumor volume was measured every 3 days and calculated using the following formula: (short diameter)^2^ × (long diameter)/2 [57].

### 4.14. Multiplex Immunofluorescence Staining of Tissues

The operation was performed according to the instructions of the four-color multi-labeling kit (TSA-rab) (panovue, Beijing, China). First, paraffin-embedded sections were incubated at 60 °C for 1 h before deparaffinization with xylene and graded ethanol hydration. Antigen retrieval was performed by boiling the slides in citric acid buffer. Subsequently, they were blocked with 5% goat serum for 10 min. Then, they were incubated with primary antibody CD4 (1:100) for 1 h at room temperature, dipped in 1xTBST buffer, and then incubated with HRP secondary antibodies for 10 min at 37℃. After removing the secondary antibody, fluorescently stained amplification signal solution (1:100 dilution) was added to the slides and incubated at room temperature for 10 min, immersed in 1xTBST buffer for washing, and then antigen retrieval was performed with citric acid buffer. After cooling to room temperature, they were washed by immersion in 1x TBST buffer. CD8 antibody staining was performed following the experimental procedure described above. Finally we stained with DAPI working solution and incubated at room temperature for 3 min in a humidified incubator. They were dipped in 1xTBST buffer for 3 min, coverslipped, mounted with glycerol, and photographed and observed with a confocal microscope.

### 4.15. Histopathology and Immunohistochemistry

Paraffin-embedded sections were incubated at 60 °C for 1 h before deparaffinization with xylene and graded ethanol hydration. Nuclei were stained with hematoxylin and cytoplasm was stained with eosin, mounted with neutral gum, and observed. As for immunohistochemistry, the slides were boiled in citric acid buffer, antigen retrieval was performed, and the activity of endogenous peroxidase was blocked with 3% H_2_O_2_. This was followed by blocking with 5% goat serum for 0.5 h. Sections were incubated overnight at 4 °C with rabbit anti-human PD-L1, IFN-γ antibodies (1:100; ABclonal, Wuhan, China), followed by 0.5 h at 37 °C with enzyme-labeled secondary antibodies. Immunohistochemical detection was performed using a DAB substrate kit (Sangon, Shanghai, China), and nuclei were counterstained with hematoxylin. Photographs were taken using a multifunction microplate detection system (BIOTEK, CYTATION5).

### 4.16. Statistical Analysis

Data were analyzed with GraphPad Prism 8. Statistical analysis was performed using two-way ANOVA or Student’s *t*-test to determine the significance of differences. *p* values < 0.05 compared with the control or indicator groups were considered statistically significant. Significance levels are presented as * *p* < 0.05, ** *p* < 0.01, *** *p* < 0.001, and **** *p* < 0.0001. All data were obtained from three independent experiments.

## 5. Conclusions

In conclusion, our study revealed that fascaplysin induced both ferroptosis and apoptosis in lung cancer cells for the first time. Furthermore, it promoted the expression of PD-L1 in NSCLC cells and enhanced the anticancer effect of PD-1 immune checkpoint inhibitors. Our study provides evidence for the clinical use of fascaplysin in combination with anti-PD-1 as an effective cancer immunotherapy.

## Data Availability

The data used to support the findings of this study are included within the article.
